# Comparative transcriptome analysis of maize (*Zea mays* L.) seedlings in response to copper stress

**DOI:** 10.1515/biol-2022-0953

**Published:** 2024-11-06

**Authors:** Mengyan Zhang, Lin Zhao, Zhenyu Yun, Xi Wu, Qi Wu

**Affiliations:** Sub-Institute of Agriculture and Food Standardization, China National Institute of Standardization, Beijing, China; Agricultural Genomics Institute at Shenzhen, Chinese Academy of Agricultural Sciences, Shenzhen, China

**Keywords:** maize, copper stress, transcriptome, differential expression analysis, functional classification, KEGG pathway

## Abstract

Copper (Cu) is considered one of the major heavy metal pollutants in agriculture, leading to reductions in crop yield. To reveal the molecular mechanisms of resistance to copper stress in maize (*Zea mays* L.) seedlings, transcriptome analysis was conducted on the hybrid variety Zhengdan 958 exposed to 0 (control), 5, and 10 mM Cu stress using RNA-seq. In total, 619, 2,685, and 1,790 differentially expressed genes (DEGs) were identified compared to 5 mM versus 0 mM Cu, 10 mM versus 0 mM Cu, and 10 mM versus 5 mM Cu, respectively. Functional categorization of DEGs according to Gene Ontology revealed that heme binding, defense response, and multiorganism processes were significantly enriched under copper stress. Additionally, Kyoto Encyclopedia of Genes and Genomes enrichment analysis suggested that the copper stress response is mediated by pathways involving phenylpropanoid biosynthesis, flavonoid biosynthesis, and glutathione metabolism, among others. The transcriptome data demonstrated that metabolite biosynthesis and glutathione metabolism play key roles in the response of maize seedlings to copper stress, and these findings provide valuable information for enhancing copper resistance in maize.

## 1 Introduction

Heavy metals are regarded as hazards to the environment and living organisms. Global agricultural soil pollution by heavy metals is a serious concern due to their impacts on crop yields and health risks throughout the food chain [1]. Among heavy metals, copper is an essential micronutrient for plants and participates in various morphological, physiological, and biochemical processes [2]. However, the use of Cu-containing herbicides, insecticides, and fungicides, and discharge from industrial wastewater and sewage sludge result in large amounts of copper entering the ecosystem [3]. A copper concentration in soil that exceeds a certain threshold inhibits the metabolic function, damages the cell membrane integrity, interferes with seed germination and seedling emergence, and even causes plant death [4,5,6]. Rice growth is severely inhibited by high soil Cu levels (300–500 mg/kg or above), and the roots are more sensitive than other parts of rice plants [7,8]. Excessive copper significantly reduces photosynthesis but enhances the lignification of cell walls, thus slowing the growth of wheat seedlings [9].

Maize (*Zea mays* L.) is an important cereal crop grown throughout the world and is also an important genetic model plant. Copper stress also affects the growth and development of maize plants through changes in plant height, germination rate, germination potential, chlorophyll content, and soluble sugar content [10]. Excess copper can also produce reactive oxygen species (ROS), inactivate enzymes, and break down protein structures, leading to phytotoxicity in maize [11,12,13]. Within the concentration range of 50–300 mg/L Cu, the maize displays a considerable tolerance of over 60% for the roots and stems. At a 400 mg/L Cu concentration, the root growth experiences a substantial 91% inhibition, while the stem growth is inhibited by 53% [14]. Cu (1 mmol/L) inhibits the activity of protective enzymes in maize seedlings and damages the structure and function of the cell membrane [15]. At the root apex of maize seedlings, Cu exposure generates oxidative damage and alters root hormonal homeostasis, leading to strong inhibition of root growth [16]. Maize seedlings are highly susceptible to stress, which directly affects agricultural production and causes considerable economic losses. In summary, the effects of Cu stress on crops are multifaceted and include physiological and biochemical reactions, changes in spectral characteristics, seed germination and defense mechanisms, growth and development, antioxidant enzyme activities, and spectral monitoring. These effects may reduce the growth rate and yield of crops; therefore, understanding these effects is highly important for both agricultural production and environmental protection.

High-throughput sequencing for transcriptome profiling is a powerful tool that has been widely used and is particularly suitable for the study of complex gene regulatory networks [17]. Transcriptomic analyses have shown that heat shock proteins, transcription factors, and secondary metabolites play important roles in the response of 21-day-old maize seedlings to heat stress [18]. Differentially expressed genes (DEGs) involved in response to cold and drought treatment during seed germination, including those in the bHLH, NAC, MYB, and WRKY families, have been identified by RNA-seq analysis, and these findings suggest that common mechanisms may participate during maize seed germination in response to different abiotic stresses [19].

Since the transcriptome response of maize seedlings to Cu stress is unclear, a bioinformatic transcriptome analysis was performed to determine the molecular response mechanism of maize seedlings in the present study. A series of genes, possibly responsible for Cu tolerance, were identified and characterized, increasing the understanding of the stress response and defense mechanisms involved. This molecular knowledge is imperative for identifying new approaches to promote tolerance to Cu stress in maize. Hence, analyzing the functional genomics underlying the mechanisms of the Cu response and identifying target genes and pathways in maize will aid in the breeding of resistant varieties.

## 2 Materials and methods

### 2.1 Culture and treatment of plant material

Seeds of the maize hybrid line “Zhengdan 958” were sterilized and imbibed in distilled water for 12 h. The imbibed seeds were germinated. The seedlings were subsequently grown in a growth cabinet under the following conditions: 25°C, 70% humidity, and a 16-h light/8/h dark cycle. Copper sulfate (CuSO_4_·5H_2_O) was used to study Cu stress. Seven-day-old seedlings were grown in Petri dishes supplemented with 0, 5, or 10 mM Cu. After 1 week, the roots of the control and Cu-treated plants were sampled for growth measurement and transcriptome sequencing, and three independent experiments were performed (CK and Cu represent the control and Cu-treated plants, respectively).

### 2.2 Growth measurements

Maize seedlings were washed three times in double distilled water before the measurements. The images of the seedlings were captured using a Nikon D90 unilateral camera and imported to ImageJ software (NIH, Bethesda, MD, USA) for quantitative analysis of the root length. The root weight was measured using an electronics analytical balance (Mettler Toledo, Zurich, Switzerland). About 30 individual seedlings from the control and the treatment groups were measured for the length and weight tests, and three repeated experiments were performed, respectively.

### 2.3 RNA isolation and library construction

Total RNA from the roots of plants treated with 0, 5, or 10 mM Cu was isolated using a TRIzol reagent kit (Invitrogen, Carlsbad, CA, USA) according to the manufacturer’s protocol. Total RNA concentration and quality were examined using a NanoDrop One spectrophotometer (NanoDrop, Wilmington, DE, USA), and the RNA was identified using RNase-free agarose gel electrophoresis. cDNA libraries were constructed and sequenced using an Illumina NovaSeq platform (Illumina, San Diego, CA, USA) by Novogene Co., Ltd. (Beijing, China).

### 2.4 Acquirement of mapped reads and quantification of gene abundance

After sequencing, the raw reads were filtered to obtain high-quality clean reads by removing reads containing adapters, reads containing poly-N sequences, and low-quality reads from the raw data. An index of the reference genome was built, and paired-end clean reads were aligned to the maize B73 reference genome using HISAT2 v2.0.5. The mapped reads were assembled using StringTie (v1.3.3b) [20].

### 2.5 Differential expression analysis

Gene expression levels were quantified using the fragments per kilobase million (FPKM) method, a standard approach for estimating the number of transcripts per gene [21]. To identify DEGs, three comparative analyses were conducted: 0 mM Cu versus 5 mM Cu, 0 mM Cu versus 10 mM Cu, and 10 mM Cu versus 5 mM Cu. Differential expression analysis was performed using the DESeq2 R package (1.20.0). DESeq2 provides statistical routines for determining differential expression in digital gene expression data using a model based on the negative binomial distribution. The resulting *p* values were adjusted using Benjamini and Hochberg’s approach for controlling the false discovery rate. Genes were deemed differentially expressed if they exhibited a fold change of at least 2 and an adjusted *p* value of 0.05 or lower, indicating a significant change in expression levels at different copper concentrations.

### 2.6 Gene ontology (GO) and Kyoto Encyclopedia of Genes and Genomes (KEGG) enrichment analyses

The GO enrichment analysis of DEGs was implemented using the GOseq R package, which corrected for the gene length bias, ensuring a more accurate representation of gene enrichment. GO terms with corrected *p* values ≤0.05 were defined as significantly enriched. The GO analysis of the DEGs revealed significant functional enrichment in three categories: molecular function (MF), biological process (BP), and cellular component (CC). Furthermore, the clusterProfiler R package was utilized to determine the statistical enrichment of DEGs in KEGG pathways.

### 2.7 Quantitative real-time PCR

Total RNA was reverse-transcribed into cDNA using a reverse transcription kit (TaKaRa, Tokyo, Japan). Quantitative real-time PCR was performed with SYBRPremix Ex Taq^TM^ II (TaKaRa, Tokyo, Japan) and monitored using a Bio-Rad CFX Connect Real-time system (Bio-Rad, CA, USA). The PCR cycle was performed with a three-step method, and the relative expression was determined using the 2^─∆∆CT^ method. GAPDH was used as the reference gene, and the details of all primers are described in Table S1. Three biological replicates were performed for each sample.

## 3 Results

### 3.1 Effects of Cu exposure on the growth and physiological properties of maize

**Figure 1:**
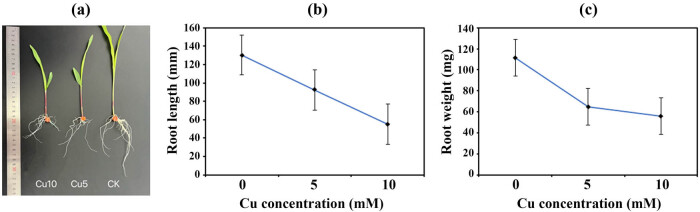
Effects of Cu exposure on the growth and physiological properties of maize. (a) Root growth status of maize seedlings treated with different Cu concentrations. (b) Root length under different Cu treatments. (c) Root weight under different Cu treatments.

After 7 days of copper treatment, maize seedlings growth slowed down significantly (Figure 1a). As shown in Figure 1b, the lengths of the roots of the maize plants treated with 5 and 10 mM Cu were approximately 29.16 and 57.79% shorter than those of the control plants, respectively. Additionally, as depicted in Figure 1c, the root weights of maize were significantly reduced after exposure to 5 and 10 mM Cu.

### 3.2 RNA sequencing and data quality assessment

**Figure 2:**
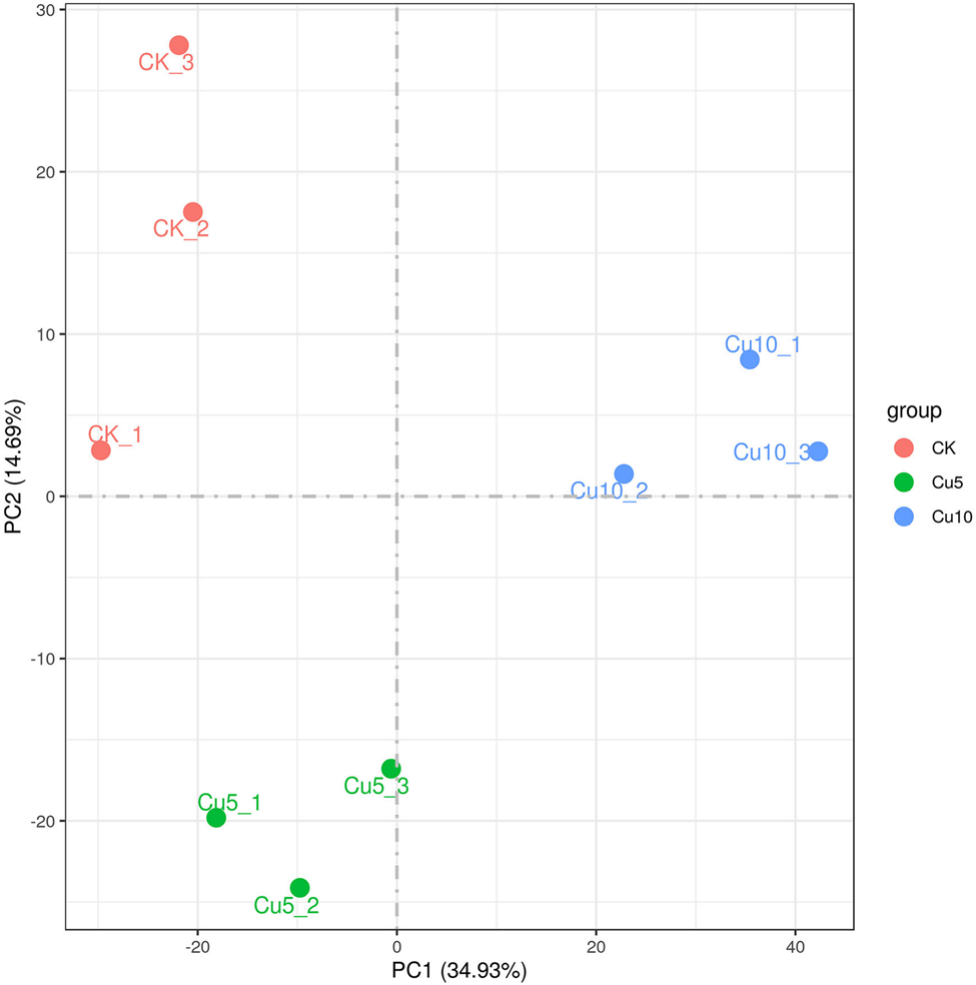
PCA plot of samples of maize seedlings grown under control and copper stress conditions. CK_1, CK_2, and CK_3 represent samples from control seedlings, whereas Cu5_1, Cu5_2, Cu5_3, Cu10_1, Cu10_2, and Cu10_3 represent the samples from seedlings exposed to copper stress.

In total, 59.6 Gb of clean reads were obtained from the transcriptomic analysis of nine samples, with the percentages of Q20 and Q30 bases exceeding 97.6 and 93.55%, respectively (Table 1). This finding indicates that the sequencing results were of high quality and appropriate for subsequent analysis. Principal component analysis (PCA) is commonly used to identify overall variance in transcriptomic data. The PCA results indicated clear differential expression among the CK, Cu5, and Cu10 groups. The first principal component accounted for 34.93% of the variance, and the second for 14.69% (Figure 2).

**Table 1:** Statistical analysis of RNA sequencing data from maize seedlings

Samples	Clean reads	Clean bases	Q20	Q30
CK_1	47515052	7.13G	97.6	93.55
CK_2	46414328	6.96G	97.88	94.28
CK_3	48318746	7.25G	97.9	94.28
Cu5_1	44183970	6.63G	97.69	93.76
Cu5_2	44398012	6.66G	97.84	94.21
Cu5_3	41727134	6.26G	97.88	94.24
Cu10_1	41292278	6.19G	97.86	94.24
Cu10_2	43739870	6.56G	97.85	94.2
Cu10_3	39702768	5.96G	97.69	93.88

### 3.3 Identification and comparison of stress-specific DEGs

**Figure 3:**
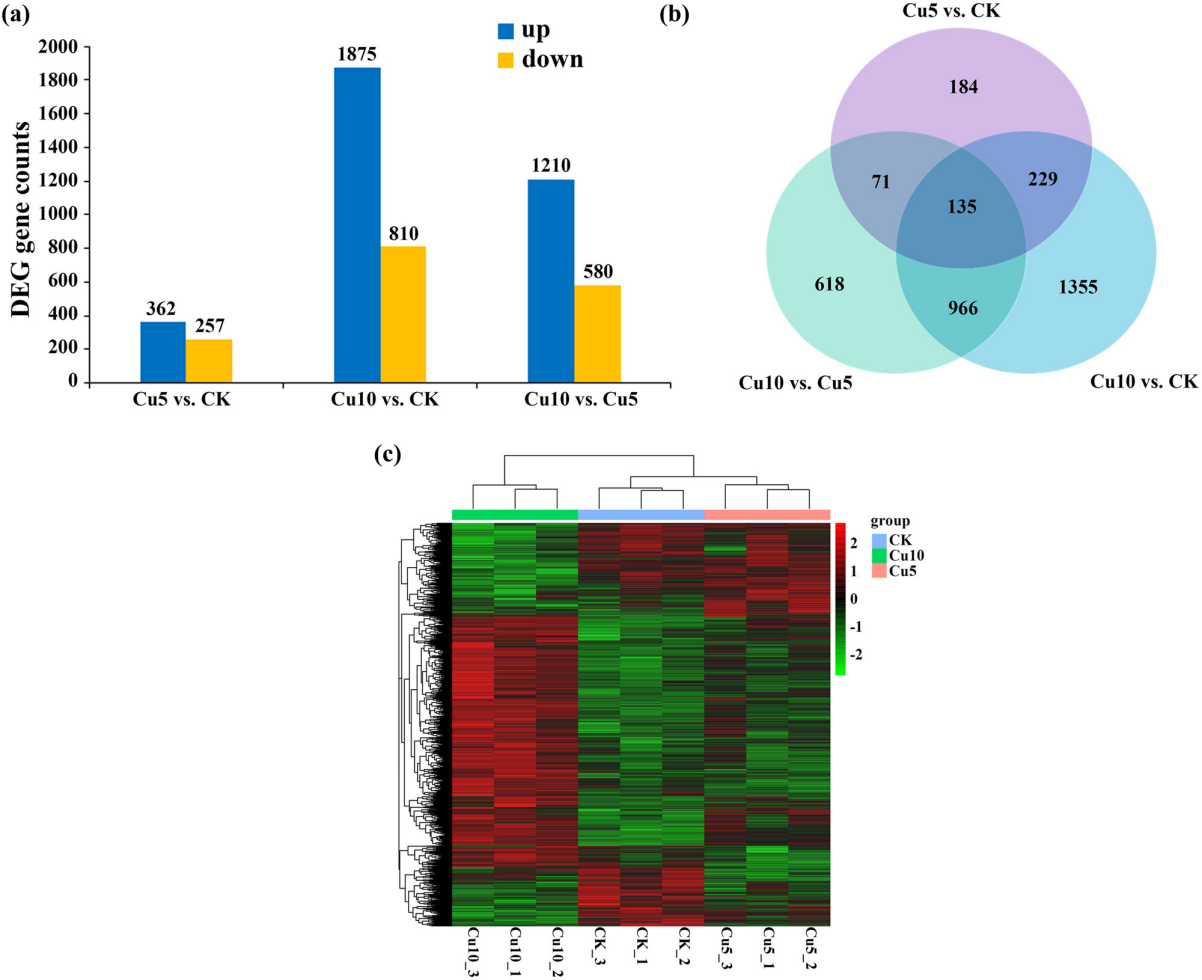
Bar chart (a), Venn diagram (b), and heatmap (c) of DEGs in maize seedlings under copper stress compared to the control seedlings. Genes that met the following criteria were considered differentially expressed: DESeq2 *p*adj ≤ 0.05 and |log2FoldChange| ≥ 1.0. (a) The *x*-axis indicates **Cu5 vs CK**, **Cu10 vs CK**, and **Cu10 vs Cu5**. The *y*-axis indicates the number of genes. The blue and yellow colors represent upregulated and downregulated genes, respectively. (b) **Cu5 vs CK**, **Cu10 vs CK**, and **Cu10 vs Cu5** comparisons. In (c), the red and green colors represent upregulated and downregulated genes, respectively.

Based on the screening criteria (fold-change ≥2 and adjusted *p* value ≤0.05), 619 DEGs (362 upregulated and 257 downregulated DEGs) were identified in the roots of maize plants treated with 5 mM Cu compared to those in the CK group. Similarly, 2,685 DEGs (1,875 upregulated and 810 downregulated) were identified in the roots of maize plants treated with 10 mM Cu compared to those in the CK group, whereas a total of 1,790 DEGs (1,210 upregulated and 580 downregulated) were found in the roots of maize plants treated with 10 mM Cu compared to those in the 5 mM Cu group (Figure 3a). According to the three abovementioned comparisons, the total number of upregulated genes was greater than that of downregulated genes. Additionally, most DEGs were identified from the **Cu10 vs CK** comparison. A Venn diagram of the DEGs revealed that 135 DEGs were identified from all three comparisons (Figure 3b). A heatmap of the hierarchical clustering analysis revealed that the DEGs in the 10 mM Cu-treated group were effectively distinguished from those in the 5 mM Cu-treated and CK groups (Figure 3c).

### 3.4 Functional classification of DEGs

**Figure 4:**
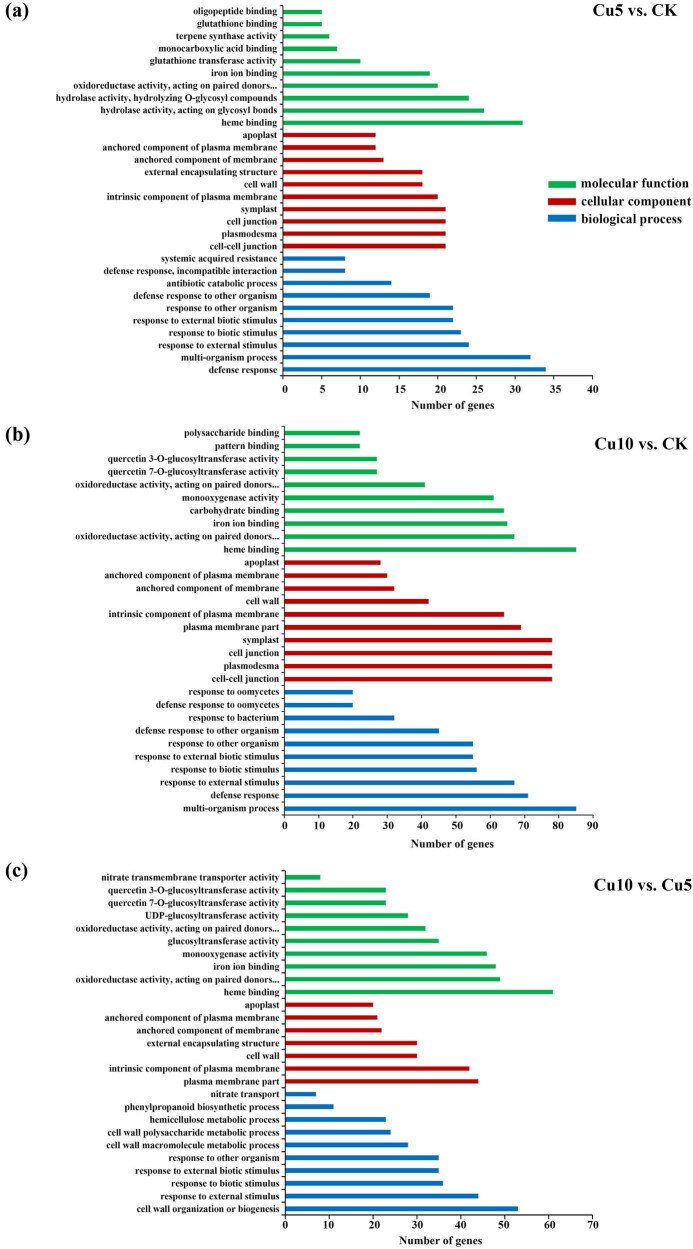
GO terms significantly enriched in DEGs. (a) **Cu5 vs CK** comparison, (b) **Cu10 vs CK** comparison, and (c) **Cu10 vs Cu5** comparison. The GO terms associated with MFs, CCs, and BPs are shown.

GO enrichment analysis results revealed the top 30 significantly enriched (*p*adj ≤ 0.05) functional terms (Figure 4). Similar terms were significantly enriched in the DEGs identified from the **Cu5 vs CK** and **Cu10 vs CK** comparisons. Heme binding was the most highly enriched term in the MF category. Symplast, cell junction, and plasmodesma were the most highly enriched terms in the CC category. Defense response and multiorganism process were the most highly enriched terms in the BP category. In the **Cu10 vs Cu5** comparison, heme binding remained the most highly enriched term in the MF category, and plasma membrane part and cell wall organization or biogenesis were the most highly enriched terms in the CC and BP categories, respectively.

### 3.5 KEGG pathway enrichment analysis of DEGs

**Figure 5:**
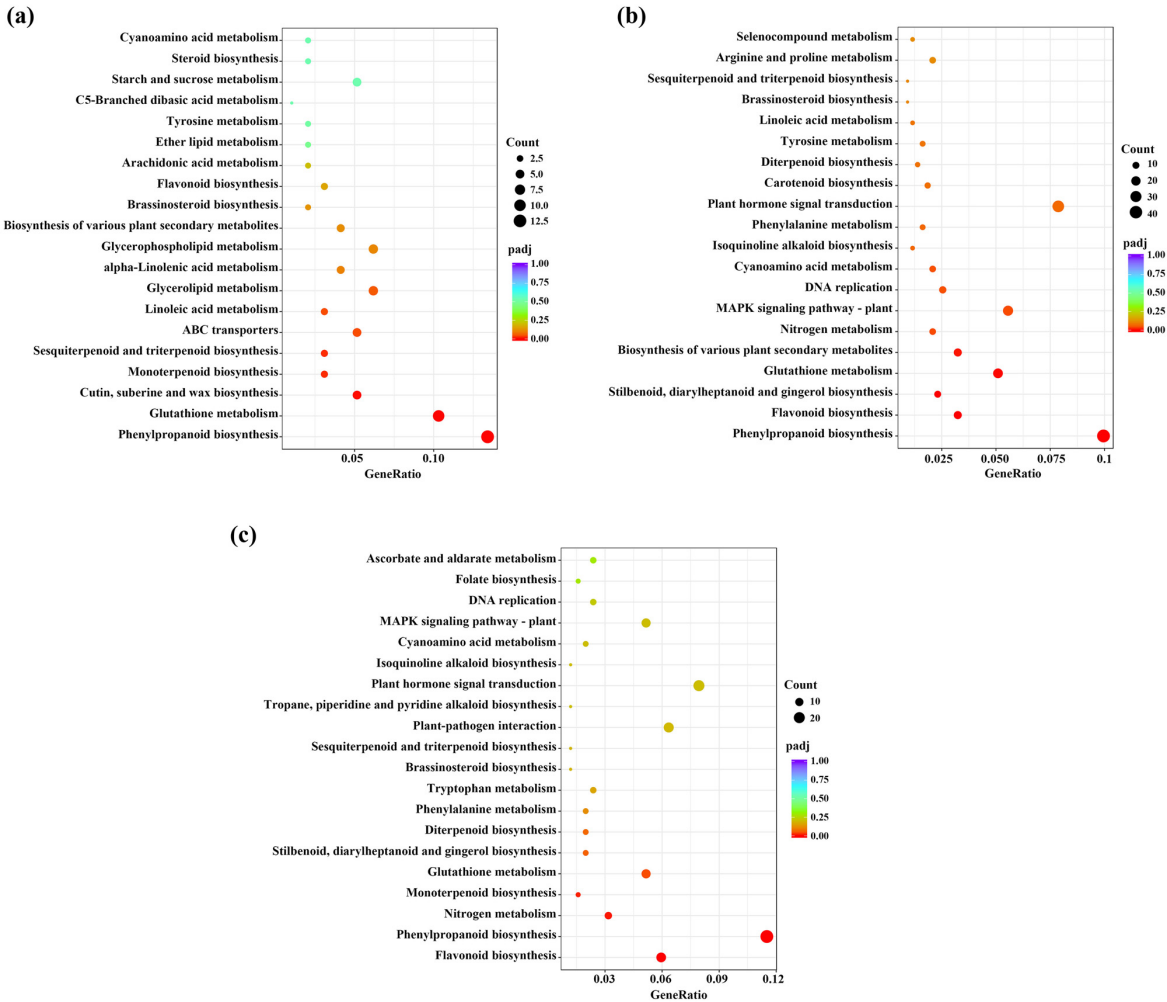
KEGG pathways significantly enriched in DEGs. (a) **Cu5 vs CK** comparison, (b) **Cu10 vs CK** comparison, and (c) **Cu10 vs Cu5** comparison.

The results from the KEGG pathway enrichment analysis revealed the most highly enriched pathways (Figure 5). Phenylpropanoid biosynthesis was the most dominant pathway in the **Cu5 vs CK** comparison, followed by glutathione metabolism; cutin, suberin, and wax biosynthesis; monoterpenoid biosynthesis; and sesquiterpenoid and triterpenoid biosynthesis. In the **Cu10 vs CK** comparison, five pathways, namely, phenylpropanoid biosynthesis, flavonoid biosynthesis, stilbenoid, diarylheptanoid and gingerol biosynthesis, glutathione metabolism, and biosynthesis of various plant secondary metabolite pathways, were significantly enriched. In addition, the flavonoid biosynthesis and phenylpropanoid biosynthesis pathways were significantly enriched in the **Cu10 vs Cu5** comparison.

### 3.6 DEGs related to phenylpropanoid and flavonoid biosyntheses

Regarding phenylpropanoid biosynthesis, we identified 24 downregulated and 5 upregulated DEGs in the **Cu10 vs Cu5** comparison, 4 upregulated and 9 downregulated DEGs in the **Cu5 vs CK** comparison, and 24 downregulated and 19 upregulated DEGs in the **Cu10 vs CK** comparison (Table 2). Most DEGs controlling phenylpropanoid biosynthesis that were identified from the **Cu10 vs CK** and **Cu10 vs Cu5** comparisons, including genes involved in peroxidase syntheses, such as prx39, IDP7923, and pox2, were downregulated. The **Cu10 vs Cu5** and **Cu10 vs CK** comparisons identified 15 upregulated DEGs and 11 upregulated and 3 downregulated DEGs related to flavonoid biosynthesis, respectively (Table 2).

**Table 2:** KEGG pathway enrichment analysis of DEGs

Pathway ID	Pathway	*p*adj	Count	Up	Down
**Cu5 vs CK**					
zma00940	Phenylpropanoid biosynthesis	0.001	13	4	9
zma00480	Glutathione metabolism	0.001	10	4	6
zma00073	Cutin, suberin, and wax biosynthesis	0.002	5	0	5
zma00902	Monoterpenoid biosynthesis	0.012	3	0	3
zma00909	Sesquiterpenoid and triterpenoid biosynthesis	0.014	3	3	0
zma02010	ABC transporters	0.039	5	2	3
zma00591	Linoleic acid metabolism	0.039	3	2	1
zma00561	Glycerolipid metabolism	0.051	6	2	4
**Cu10 vs CK**					
zma00940	Phenylpropanoid biosynthesis	0.000	43	19	24
zma00941	Flavonoid biosynthesis	0.000	14	11	3
zma00945	Stilbenoid, diarylheptanoid and gingerol biosynthesis	0.001	10	9	1
zma00480	Glutathione metabolism	0.001	22	15	7
zma00999	Biosynthesis of various plant secondary metabolites	0.004	14	8	6
zma00910	Nitrogen metabolism	0.037	9	2	7
zma04016	MAPK signaling pathway – plant	0.037	24	19	5
zma03030	DNA replication	0.042	11	0	11
zma00460	Cyanoamino acid metabolism	0.043	9	5	4
zma00950	Isoquinoline alkaloid biosynthesis	0.068	5	5	0
zma00360	Phenylalanine metabolism	0.068	7	7	0
zma04075	Plant hormone signal transduction	0.070	34	26	8
zma00906	Carotenoid biosynthesis	0.079	8	2	6
zma00904	Diterpenoid biosynthesis	0.080	6	5	1
zma00350	Tyrosine metabolism	0.086	7	7	0
zma00591	Linoleic acid metabolism	0.086	5	3	2
**Cu10 vs Cu5**					
zma00941	Flavonoid biosynthesis	0.000	15	15	0
zma00940	Phenylpropanoid biosynthesis	0.000	29	5	24
zma00910	Nitrogen metabolism	0.005	8	1	7
zma00902	Monoterpenoid biosynthesis	0.009	4	4	0
zma00480	Glutathione metabolism	0.039	13	10	3
zma00904	Diterpenoid biosynthesis	0.070	5	5	0

### 3.7 DEGs related to glutathione metabolism

In the present study, we identified 3 downregulated and 10 upregulated DEGs related to glutathione metabolism in the **Cu10 vs Cu5** comparison, 6 downregulated and 4 upregulated DEGs related to glutathione metabolism in the **Cu5 vs CK** comparison, and 7 downregulated and 15 upregulated DEGs related to glutathione metabolism in the **Cu10 vs CK** comparison. Most of these genes were upregulated in the **Cu10 vs CK** and **Cu10 vs Cu5** comparisons but downregulated in the **Cu5 vs CK** comparison, suggesting that maize seedlings suffered more oxidative stress during exposure to high Cu concentrations (Table 2).

### 3.8 qRT–PCR verification

**Figure 6:**
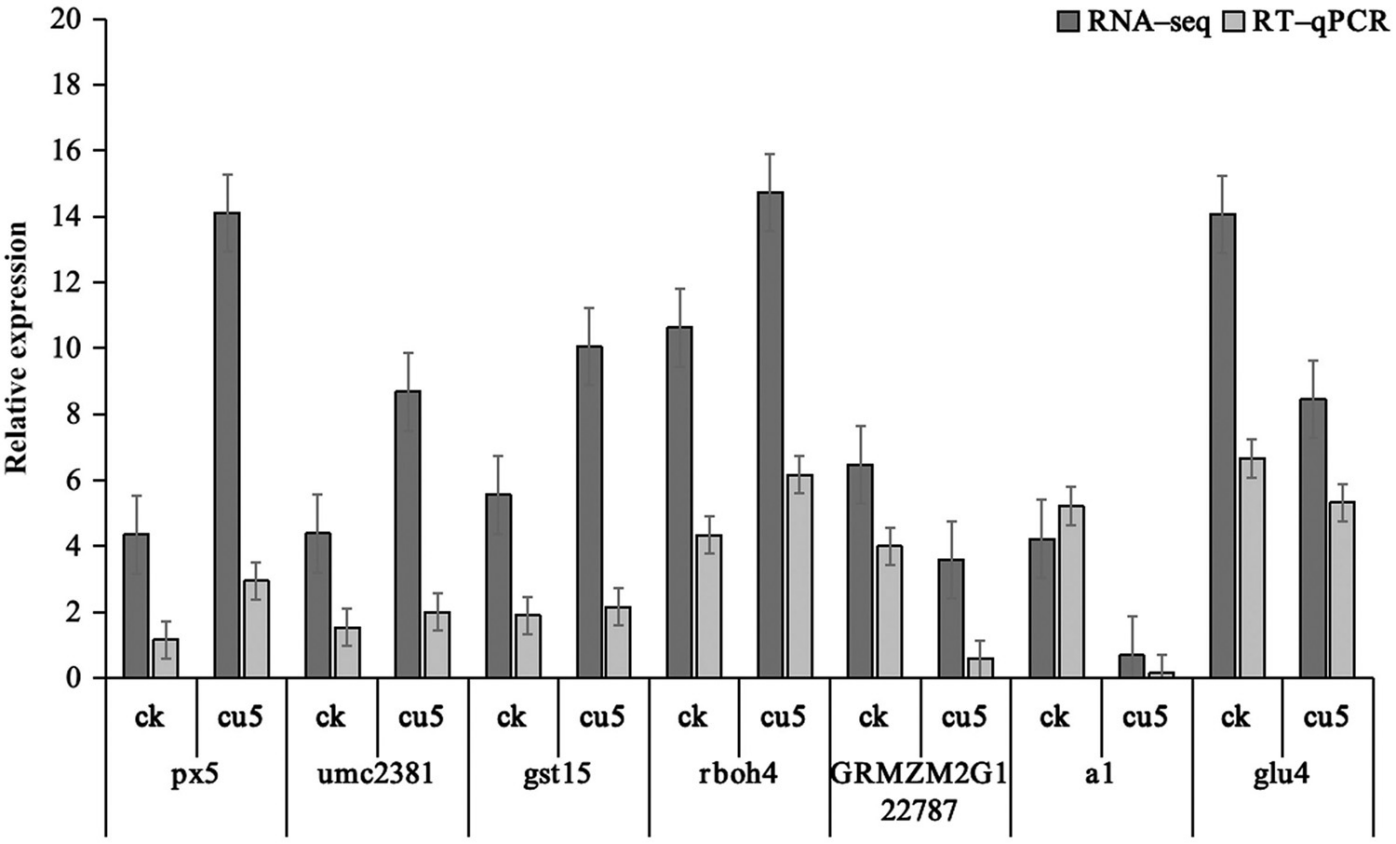
Validation of the expression of genes in maize seedlings using qRT‒PCR. The error bars represent the standard deviations of three independent biological replicates. A value of *p* < 0.05 indicates a statistically significant difference.

Seven DEGs involved in phenylpropanoid biosynthesis, flavonoid biosynthesis, and glutathione metabolism were randomly selected for qRT‒PCR analysis to verify the reproducibility of the gene expression data obtained by RNA-seq analysis. As shown in Figure 6, the expression patterns of these DEGs (*px5*, *umc2381*, *gst15*, *rboh4*, *GRMZM2G122787*, *a1,* and *glu4*) were consistent with the FPKM values obtained from RNA-seq, which confirms the effectiveness of DEGs in this study.

## 4 Discussion

Copper pollution has attracted extensive attention. Crops suffering from copper stress show toxic symptoms, which subsequently result in poor seed germination, stunted growth, and decreased yield [22]. Maize seedlings are unable to tolerate 1 mM Cu, and this concentration inhibits the root growth and activities of protective enzymes in maize seedlings [15]. At the concentrations tested in this study, toxic effects were detected in the roots of the maize seedlings (Figure 1). The abovementioned toxicity symptoms can lead to reduced agricultural productivity, which has economic and food security implications.

With the development of high-throughput sequencing technology, RNA-seq technology has been successfully applied to transcriptome analysis of rice [23], wheat [24], maize [25], and other crops [26]. Specifically, this study enhances our comprehension of the molecular mechanisms underlying maize’s reaction to copper stress. A total of 23,413 genes were identified by transcriptome analysis in maize seedlings under different conditions. Further, the identification of DEGs (619, 2,685, and 1,790) in different copper concentration comparisons (**Cu5 vs CK**, **Cu10 vs CK**, and **Cu10 vs Cu5**) suggested that maize seedlings exhibit a range of responses to varying levels of copper stress (Figure 3a). We found that the DEGs in the 10 mM Cu-treated group were effectively distinguished from those in the 5 mM Cu-treated and CK groups (Figure 3c), suggesting that the effect of high Cu concentration on gene expression patterns was more obvious. The GO enrichment analysis of the DEGs reveals a consistent enrichment pattern in **Cu5 vs CK** and **Cu10 vs CK** comparisons, including heme binding, cell–cell junction, and defense response, which contrast with the **Cu10 vs Cu5** comparison (Figure 4). These results indicate that the physiological and molecular responses exhibit significant variation as the concentration of Cu increases.

Glutathione is a low molecular weight tripeptide compound that contains a sulfhydryl group and is an important metal chelator and antioxidant [27], which protects cells, retains enzyme activity and protein function, and prevents damage to the cytoplasm and outer membrane [28,29]. Glutathione is also an important cofactor for glutathione glycol peroxidase, glyceraldehyde 3-phosphate dehydrogenase, glyoxalase, and triose dehydrogenase [30]. In addition, glutathione is involved in the formation of disulfide bonds in proteins and the maintenance of the transport of amyloid across cell membranes [31,32]. In the present study, we isolated 28 genes involved in glutathione metabolism from the **Cu5 vs CK**, **Cu10 vs CK**, and **Cu10 vs Cu5** comparisons. Most of the upregulated genes, such as *gst5*, *gst12*, *gst15*, *gst23, gst31,* and *gst35*, were related to the synthesis of glutathione S-transferase (GST). Due to the high affinity of GST to the sulfhydryl group, it shows strong detoxification ability. GST is also a key enzyme in glutathione metabolism, catalyzing the initial steps of this pathway. High levels of copper stress increase the expression levels of GST-related genes in maize, and the GST activity in maize may increase during exposure to copper stress. Modulation of glutathione metabolism in response to copper stress suggests that these genes and their associated metabolic pathways may be targets for genetic improvement to enhance copper tolerance in maize. Collectively, these findings elucidate the complex interplay between glutathione metabolism and plant resistance to environmental stress, providing valuable insights for future research aimed at improving crop resistance to heavy metal toxicity.

Phenylpropanoids are specialized metabolites derived from phenylalanine. In plants, phenylpropanoid biosynthesis produces a variety of aromatic metabolites with important biological functions [33]. Peroxidase catalyzes redox reactions to produce ROS during phenylpropanoid biosynthesis. ROS are important signaling molecules during normal plant growth and development of plants. ROS-related redox signals play a crucial role in plant growth and development as well as adaptation to the environment, controlling almost all physiological processes of cells, such as gene expression and translation, metabolism, and death due to aging [34]. The KEGG pathway enrichment analysis revealed that phenylpropanoid biosynthesis was the most significant enrichment pathway among the DEGs identified from the **Cu5 vs CK**, **Cu10 vs CK**, and **Cu10 vs Cu5** comparisons (Figure 5). Downregulation of peroxidase genes, including *pox2*, *px17*, *px24*, *prx39,* and *prx69*, may have a significant impact on phenylpropanoid biosynthesis and maize root growth, which provides valuable insights into potential targets for genetic modification to enhance stress tolerance and increase seed germination rate.

Flavonoids are important secondary metabolites formed in the long-term ecological adaptation process of plants, which are used to resist invasion of harsh ecological conditions, animals, and microorganisms [35,36,37]. These basic carbon skeleton compounds are widely distributed in plants. Flavonoids exhibit tissue, developmental, and environmental factor specificity in plant metabolism, participate in plant ecological defense, serve as messengers in reproductive processes, and play an important role in plant growth and stress responses [38]. Previous studies have shown that flavonoids have multiple functions; for example, flavonoids accumulate and have antioxidant abilities, thereby improving maize plant adaptability to abiotic stress [39]. In this study, the expression of several key genes (*a1*, *chi6*, *hct9*, *fomt4*, *fht1,* and *umc2381*) involved in flavonoid biosynthesis showed significant differences in maize seedlings before and after Cu stress, suggesting that flavonoids may be related to Cu stress. Transcriptome analysis was used to annotate upregulated genes related to flavonoid biosynthesis, including chalcone-flavanone isomerase family protein, flavonoid 3′-monooxygenase, anthranilate *N*-benzoyltransferase protein, cytochrome P45, spermidine hydroxycinnamoyl transferase, and hydroxycinnamoyltransferase. This upregulation suggests that plants synergistically increase their flavonoid production under copper stress, which may help mitigate oxidative damage and protect plants from the toxic effects of copper. In summary, this study provides evidence that flavonoids are dynamically regulated in maize responses to copper stress, highlighting their critical role in plant detoxification and defense against heavy metal poisoning.

## 5 Conclusion

In conclusion, transcriptome studies of maize seedling roots provide data on their tolerance mechanisms to copper stress. Through RNA-seq data analysis, we identified 619, 2,685, and 1,790 DEGs in the **Cu5 vs CK**, **Cu10 vs CK** and **Cu10 vs Cu5** comparisons, respectively. By integrating the current findings and existing data, copper stress was shown to affect the metabolism and biosynthesis of various plant compounds. Although the exact roles of the candidate genes involved in these pathways remain to be elucidated, the results of this study provide valuable information for further analysis of the response mechanism to copper stress.

## Supplementary Material

Supplementary Table
