# Supplementary material for: Comparative transcriptome analysis of maize (Zea mays L.) seedlings in response to copper stress
Source: Open Life Sci. 2024 Nov 6;19(1):20220953. doi: 10.1515/biol-2022-0953 (PMC11554555; doi:10.1515/biol-2022-0953)
Supplement: Supplementary Table [file biol-2022-0953-sm.pdf]

# Supplementary material

**Table S1:** All specific primer sequences

| Primer name | Sequence (5′–3′) | Primer type | Method |
|---|---|---|---|
| *GAPDH* | CTGGTGCTGGAATTGCACTG | Forward | RT-qPCR |
| | TCAATGACGCGGTTGCTGTA | Reverse | RT-qPCR |
| *px5* | ACTTCGCCTCCAAGAACCTCAG | Forward | RT-qPCR |
| | CGGTCGCCTGTGTTGTTAATTCC | Reverse | RT-qPCR |
| *umc2381* | TCTATTGGCTACGGCATCTTGAATC | Forward | RT-qPCR |
| | CGGAGTGGTGGTAGGTAGTTGG | Reverse | RT-qPCR |
| *gst15* | CAAGAAGGTGGCGGAGTTCATAC | Forward | RT-qPCR |
| | CAGGCACATTAGGCAGGCTTG | Reverse | RT-qPCR |
| *rboh4* | GGTAACTCCGTCAACAGCCTTTC | Forward | RT-qPCR |
| | GAGAGGAAGAAGCATTTGGTTATTTGG | Reverse | RT-qPCR |
| *GRMZM2G122787* | ATGATGACAGCGGACCAGGAC | Forward | RT-qPCR |
| | CGACATCACGACGACGGAATTG | Reverse | RT-qPCR |
| *a1* | CTGTCCAAAGACCCTGAGAATGAG | Forward | RT-qPCR |
| | GCGGAGGAAGTGAAGACGATG | Reverse | RT-qPCR |
| *glu4* | CTCCTGTTGTTGTGTCTTGTTGTTG | Forward | RT-qPCR |
| | CATCAACGACAGACTAGCACATCC | Reverse | RT-qPCR |
